# CYP3A7*1C allele is associated with reduced levels of 2-hydroxylation pathway oestrogen metabolites

**DOI:** 10.1038/bjc.2016.432

**Published:** 2017-01-10

**Authors:** Deepti Sood, Nichola Johnson, Pooja Jain, Alexandros P Siskos, Mark Bennett, Clare Gilham, Marta Cecilia Busana, Julian Peto, Isabel dos-Santos-Silva, Hector C Keun, Olivia Fletcher

**Affiliations:** 1 Faculty of Medicine, Department of Surgery and Cancer, Imperial College, London SW7 2AZ, UK; 2 Breast Cancer Now Toby Robins Research Centre, The Institute of Cancer Research, 237 Fulham Road, London SW7 3RP, UK; 3 Department of Life Sciences, Imperial College, London SW7 2AZ, UK; 4 Department of Non-Communicable Disease Epidemiology, The London School of Hygiene and Tropical Medicine, London WC1E 7HT, UK

**Keywords:** CYP3A7*1C allele, oestrogen metabolites, breast cancer

## Abstract

**Background:**

Endogenous sex hormones are well-established risk factors for breast cancer; the contribution of specific oestrogen metabolites (EMs) and/or ratios of specific EMs is less clear. We have previously identified a CYP3A7*1C allele that is associated with lower urinary oestrone (E_1_) levels in premenopausal women. The purpose of this analysis was to determine whether this allele was associated with specific pathway EMs.

**Methods:**

We measured successfully 12 EMs in mid-follicular phase urine samples from 30 CYP3A7*1C carriers and 30 non-carriers using HPLC-MS/MS.

**Results:**

In addition to having lower urinary E_1_ levels, CYP3A7*1C carriers had significantly lower levels of four of the 2-hydroxylation pathway EMs that we measured (2-hydroxyestrone, *P*=1.1 × 10^−12^; 2-hydroxyestradiol, *P*=2.7 × 10^−7^; 2-methoxyestrone, *P*=1.9 × 10^−12^; and 2-methoxyestradiol, *P*=0.0009). By contrast, 16α-hydroxylation pathway EMs were slightly higher in carriers and significantly so for 17-epiestriol (*P*=0.002).

**Conclusions:**

The CYP3A7*1C allele is associated with a lower urinary E_1_ levels, a more pronounced reduction in 2-hydroxylation pathway EMs and a lower ratio of 2-hydroxylation:16α-hydroxylation EMs in premenopausal women. To further characterise the association between parent oestrogens, EMs and subsequent risk of breast cancer, characterisation of additional genetic variants that influence oestrogen metabolism and large prospective studies of a broad spectrum of EMs will be required.

Endogenous sex hormones are well-established risk factors for breast cancer. Pooled analyses of data from prospective studies have estimated that a doubling of circulating estradiol (E_2_), free E_2_ or E_1_ is associated with a 20–30% or 30–50% increase in breast cancer risk in pre- and postmenopausal women, respectively (Key *et al*, 2002, 2013). The contribution of specific oestrogen metabolites (EMs) to breast cancer risk is less clear. Briefly, interconversion between the parent oestrogens, E_2_ and E_1_ occurs by reversible oxidation at the 17α-position of the steroid ring; conversion of parent oestrogens to EMs is by irreversible hydroxylation at the 2-, 4- or 16-positions (Figure 1; Badawi *et al*, 2001; Tsuchiya *et al*, 2005; Samavat and Kurzer, 2015). A recent review, summarising evidence from four prospective studies of oestrogen metabolism and breast cancer risk concluded that there was consistent evidence that enhanced 2-hydroxylation was associated with a reduction in risk of breast cancer that was independent of the strong positive associations of unconjugated parent oestrogens (E_2_ and E_1_) with breast cancer risk (Ziegler *et al*, 2015).

In an analysis of single-nucleotide polymorphisms (SNPs) tagging genes that are involved in oestrogen synthesis and metabolism, we identified one SNP, rs10273424, which was associated with a 22% reduction in levels of urinary E_1_ glucuronide (E_1_G) in premenopausal women (Johnson *et al*, 2012). rs10273424 maps to the cytochrome P450 3A (*CYP3A*) gene cluster at 7q22.1; the *CYP3A* genes (*CYP3A5*, *CYP3A7* and *CYP3A4*) encode enzymes that catalyse the oxidative metabolism of a wide range of exogenous and endogenous substrates including parent oestrogens (E_1_ and E_2_; Figure 1). The metabolic capacity of the CYP3A enzymes differ, depending on the substrate (Williams *et al*, 2002); with respect to oestrogen metabolism specifically, 2-hydroxylation of E_1_ to 2-OHE_1_ is catalysed by CYP3A4, 4-hydroxylation to 4-OHE_1_ is catalysed by CYP3A4 and CYP3A5 and 16α-hydroxylation to 16α-OHE_1_ is catalysed by CYP3A4, CYP3A5 and CYP3A7 (Figure 1; Badawi *et al*, 2001; Lee *et al*, 2003; Tsuchiya *et al*, 2005). Fine-mapping of the 7q22.1 association signal for urinary E_1_G levels implicated the CYP3A7*1C allele as the causal allele (Johnson *et al*, 2016). This allele, which comprises seven highly correlated single base changes mapping to the *CYP3A7* promoter, results in expression of the fetal *CYP3A7* gene in adult carriers of the CYP3A7*1C allele (Gonzalez, 1988; Kuehl *et al*, 2001; Burk *et al*, 2002).

The purpose of this current analysis was to determine whether, in addition to the association between CYP3A7*1C carrier status and lower urinary E_1_G levels, the CYP3A7*1C allele was associated with a reduction in levels of specific pathway EMs.

## Materials and methods

### Study population

The study population from which the women we included in this analysis were drawn has been described previously (Johnson *et al*, 2012). Briefly, they comprised 729 premenopausal women who were first-degree relatives and friends of breast cancer cases participating in the British Breast Cancer study (Johnson *et al*, 2005) or participants in the intervention arm of a trial of annual mammographic screening in young women conducted in Britain (Mammography Oestrogens and Growth Factors Study; Walker *et al*, 2009). To be eligible, women had to be having regular menstrual cycles, not using hormone replacement therapy or oral contraceptives and not to have been diagnosed with breast cancer at recruitment to the study. All women were of self-reported White British ancestry. To be included in the original analysis and this subsequent analysis, women had to have provided serial urine samples (six follicular phase and one luteal phase), at pre-specified days of their menstrual cycle for measurement of creatinine adjusted urinary E_1_G using an in-house enzyme-linked immunosorbent assay (Johnson *et al*, 2012). To maximise the statistical efficiency of this analysis of the CYP3A7*1C allele, which has a minor allele frequency (MAF) of just 4% in Northern European populations (Johnson *et al*, 2016), we selected 60 women on the basis of genotype; a random sample of 30 CYP3A7*1C carriers and 30 CYP3A7*1C non-carriers. We further selected the two periovulatory samples (samples three and four of the six sequential follicular phase samples) on the basis that oestrogen levels would be at their peak in these samples and this would maximise any differences in levels between CYP3A7*1C carriers and non-carriers. To minimise random variation, we used the average of these two sequential samples as our outcome variable.

### Ethics

The study was conducted in accordance with the tenets of the Declaration of Helsinki and all participants provided written informed consent.

### Genotyping

Genotyping of the tag SNP rs45467892, which is perfectly correlated with the CYP3A7*1C allele, has been described previously (Johnson *et al*, 2016). Briefly, genotyping was by Taqman (Life Technologies, Paisley, UK). The call rate was 96.9% and concordance between duplicate samples was 100%.

### HPLC-MS/MS analysis

LC-MS/MS analysis was carried out for 14 EMs using the method of (Xu *et al*, 2005, 2007). We were unable to measure one of the 15 EMs measured by (Xu *et al*, 2005, 2007) (16-Epiestriol), as there was no commercially available standard for this EM.

Briefly, two aliquots of frozen urine per woman were sent to the Mass Spectroscopy Facility for Quantitative Analysis, Faculty of Natural Science, Imperial College, London, for analysis. Hydrolysis of glucuronide and sulphate-conjugated EMs to form free EMs was carried out by mixing 500 *μ*l of freshly thawed urine with 20 *μ*l of internal standards (comprising 2 ng of each of five deuterium-labelled oestrogen metabolites (d-EMs); 17β-estradiol-d4, estriol-d3, 2-hydroxy-17β-estradiol-d5, 2-methoxy-17bestradiol-d5 and 16-epiestriol-d3; Qmx Laboratories Ltd, Dunmow, UK) and 500 *μ*l of freshly prepared enzymatic hydrolysis buffer (100 *μ*l of β-glucuronidase from *Helix pomatia* (Type H-2; Sigma-Aldrich, St Louis, MO, USA) in 10 ml 0.15 M sodium acetate buffer, pH 4.6 containing 2 mg of ascorbic acid). Samples were incubated at 37 °C overnight before extraction with dichloromethane and dansyl chloride dervitazation. The final derivatised samples (200 *μ*l) were transferred to HPLC vials. Urine samples were randomly allocated to one of six analytical batches. Each batch contained 1 matrix blank, 1 matrix blank spiked with internal standards, 8-point calibration standards, 3 quality control (QC) samples and 20 urine samples. QC samples were prepared using charcoal stripped human urine (Golden West Biological Inc., Temecula, CA, USA) with no detectable levels of oestrogen metabolites, spiked with all 14 EMs at a concentration of 2 ng ml^−1^.

Samples (10 μl) were then analysed by HPLC-electrospray ionisation/MS-MS using an Agilent 1100 HPLC coupled to an SCIEX QTRAP 6500 mass spectrometer (AB Sciex LLC, Framingham, MA, USA) running in multiple reaction monitoring (MRM) mode. Chromatographic separation was carried out on a Phenomenex Synergi Hydro-RP 4 μm × 150 mm × 2.0 mm column, at 40 °C. The solvent gradient used was 35%A (99.8% H_2_O: 0.2% CHOOH) to 85%B (99.8% MeOH: 0.2% CHOOH) over 60 min. Solvent B was held at 85% for 4 min, then the solvent returned to 35% A for 10 min equilibration before the next injection. The solvent flow rate was 250 μl min^−1^. The ESI source (type: Ion Drive Turbo V) parameters were set to the following: TEM 500 °C, Curtain Gas 45 psi, GS1 50 psi, GS2 60 psi and MS parameters were CAD gas Medium, DP 80, EP 10, CE 45 and CXP 5. A scheduled detection method was used and the MRM detection window was 120 s with a target scan time of 1 s. Transitions and retention times are listed in Supplementary Table 1.

Analyst 1.6.2 software (AB Sciex LLC) was used for quantification of the EMs. Peak quantifications were carried out using d-EM internal standards and constructing matrix matched (charcoal stripped human urine) eight-point calibration curves for each of the six analytical batches. The calibration curves were evaluated by plotting the peak area ratios of dansyl-EM/d-dansyl-EM against concentration (ng ml^−1^) of EMs in the standard and using linear regression with 1/*X* weighting to fit the data. Using this linear function, the amounts of EMs in the urine sample were interpolated.

The intra- and inter-batch coefficients of variation (CVs), evaluated from three QC samples per analytical batch, in six independent consecutive batches (*N*=18 QC samples) ranged from 6% to 10% (intra-batch CV) and 6% to 14% (inter-batch CV; Supplementary Table 2). The two highest inter-batch CVs were for 4-methoxyestrone (14%) and 4-methoxyestradiol (13%), the two oestrogen metabolites that had the lowest concentrations and which were subsequently excluded from the analysis. The LLOQ was estimated as 80 pg ml^−1^, where the intra- and inter-batch precision of all the EMs were consistently <10% and the intra- and inter batch accuracies were between 97 and 105%. The LOD for the entire assay (all 14 EMs) was estimated to be 8 pg ml^−1^.

### Statistical analysis

EMs were converted from ng ml^−1^ of urine to pg mg^−1^ creatinine using the molecular weight of the unconjugated form of each of the EMs and the amount of creatinine (measured in mg ml^−1^) for each of the samples. This allowed us to create pathway variables as described by Faupel-Badger *et al* (2010). Where both of the two samples per woman were measured successfully, we used the mean of the two measurements; where one of two measurements from a woman was missing, we used the one available measurement; where both measurements were missing, we excluded the woman from the analysis of this EM. For the majority of EMs, there were no missing values. For four EMs (2-hydroxyestrone (2-OHE1), 2-methoxyestradiol (2-MeOE_2_), 2-hydroxyestrone-3-methyl ether (3-MeOE_1_) and 16-ketoestradiol (16-ketoE_2_)), there were <10% missing values (Supplementary Table 3). For two EMs (4-methoxyestrone (4-MeOE_1_) and 4-methoxyestradiol (4-MeOE_2_)), levels were undetectable in the majority of samples (92 (76.7%) and 107 (89.2%) for 4-MeOE_1_ and 4-MeOE_2_, respectively). These two EMs were excluded from further analysis. For the 12 EMs that we were able to measure, we estimated the within-woman variation based on the two sequential samples per woman; intra-class correlation coefficients for individual EMs and grouped EMs are shown in Supplementary Table 4.

For individual and pathway EMs, we calculated geometric means and 95% confidence intervals (CIs) on the natural logarithm scale and exponentiated the values back to the original scale. Linear regression models of the log_e_-transformed EMs were used to estimate per cent differences between CYP3A7*1C carriers and non-carriers for individual and pathway EMs. We carried out unadjusted analyses and analyses that were adjusted for measurement batch (1–6), body mass index (BMI; quartiles), age at first full-term pregnancy (quartiles) and parity (0, 1–2, >2). Adjustment for these covariates did not alter effect sizes substantially and, therefore, unadjusted results are presented. We used *t*-tests of the linear regression coefficient to estimate *P*-values. We applied a Bonferroni correction to establish a statistical significance level of *P*<0.003, based on our measuring of 14 individual EMs and 5 grouped EMs. Statistical analyses were carried out using R (version 3.2.3; http://cran.r-project.org). All reported *P*-values are two-sided.

## Results

**Figure 2:**
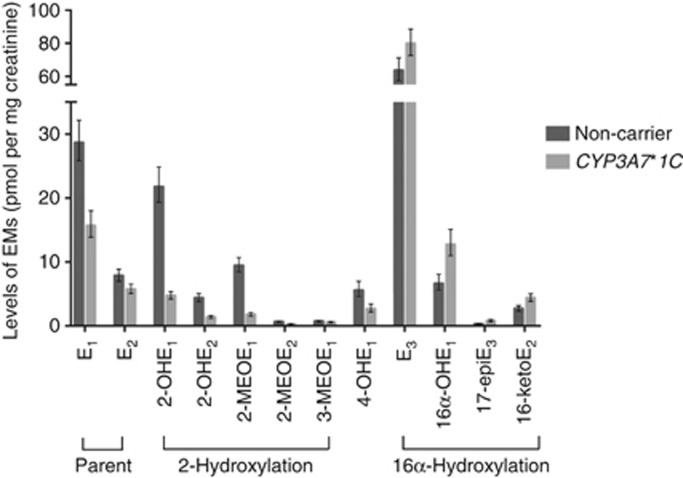
Geometric mean levels (pmol mg^−1^ creatinine) of 12 EMs that we measured in urine samples from 60 premenopausal women; 30 carriers of the CYP3A7*1C allele (light grey) and 30 non-carriers (dark grey). Estimates are the average of two samples per woman, taken on sequential days, calculated to be at or around ovulation based on the woman's usual menstrual cycle length. Error bars represent s.e. Levels of two of the 4-hydroxylation pathway EMs (4-MeOE_1_ and 4-MeOE_2_) were below detection in 92 (4-MeOE_1_) and 107 (4-MeOE_2_) of the samples analysed. These two EMs were, therefore, excluded.

Characteristics of the 60 women who were included in this analysis are presented in Table 1; there were no differences between CYP3A7*1C carriers and non-carriers. Levels of EMs in CYP3A7*1C carriers and non-carriers are shown in Figure 2 (full details of individual EMs, grouped EMs and ratios of EMs are in Supplementary Table 3). The predominant oestrogen/EMs were estriol (E_3_), E_1_ and 2-hydroxyestrone (2-OHE_1_) with geometric mean concentrations in non-carriers of 63.6, 28.6 and 21.8 pmol mg^−1^ creatinine, respectively. The least abundant EMs were the methylated 2-catechol EMs, 2-methoxyestradiol (2-MeOE_2_) and 2-Hydroxyestrone-3-methyl ether (3-MeOE_1_) and 17-Epiestriol (17-epiE_3_) with geometric mean concentrations in non-carriers of 0.67, 0.74 and 0.31 pmol mg^−1^ creatinine, respectively. Levels of the methylated 4-catechol EMs, 4-methoxyestrone (4-MeOE_1_) and 4-methoxyestradiol (4-MeOE_2_) were below the limits of detection in 92 (76.7%) and 107 (89.2%) of the 120 samples that we assayed.

Comparing CYP3A7*1C carriers with non-carriers, levels of the parent oestrogen E_1_ were 45.3% lower in CYP3A7*1C carriers (*P*=0.0005, Table 2). For the two catechol EMs (2-OHE_1_ and 2-OHE_2_) and the methylated two catechol EMs (2-MeOE_1_ and 2-MeOE_2_), the reduction in urinary levels was more extreme; compared with non-carriers, levels in CYP3A7*1C carriers were −78.3% (*P*=1.1 × 10^−12^), −67.9% (*P*=2.7 × 10^−7^), −81.2% (*P*=1.9 × 10^−12^) and −62.8% (*P*=0.0009) for 2-OHE_1_, 2-OHE_2_, 2-MeOE_1_ and 2-MeOE_2_, respectively (Table 2). By contrast, levels of the 16-pathway EMs were slightly higher in CYP3A7*1C carriers, that is, +25.5% (*P*=0.11), +91.6% (*P*=0.007) and +160.1% (*P*=0.002) higher for E_3_, 16α-hydroxyestrone (16α-OHE_1_) and 17-epiE_3_, respectively (Table 2). Adjusting for measurement batch, BMI, age at first full-term pregnancy and parity did not alter these results substantially (Supplementary Table 5).

EMs from the three different oestrogen metabolism pathways (2-hydroxylation, 4-hydroxylation and 16α-hydroxylation) have been associated with different oestrogenic and genotoxic properties (Faupel-Badger *et al*, 2010; Ziegler *et al*, 2015) with a high 2-hydroxylation:16α-hydroxylation pathway ratio, generally being considered to be associated with a reduction in breast cancer risk. Comparing CYP3A7*1C carriers with non-carriers, the 2-OHE_1_:16α-OHE_1_ ratio in carriers (0.39, 95% CI: 0.26–0.57) was significantly lower than the ratio in non-carriers (3.86, 95% CI: 2.53–5.89; *P*=1.0 × 10^−11^; Supplementary Table 3). Similarly, combining all 2-hydroxylation pathway and 16α-hydroxylation pathway metabolites, the ratio in CYP3A7*1C carriers (0.10, 95% CI: 0.07–0.13) was much lower than the ratio in non-carriers (0.51, 95% CI: 0.38–0.67; P=1.7 × 10^−9^; Supplementary Table 3).

## Discussion

To our knowledge, comprehensive data on urinary EMs in premenopausal women, measured using LC-MS, have been previously reported in three studies (Eliassen *et al*, 2009, 2012; Faupel-Badger *et al*, 2010; Maskarinec *et al*, 2012). The first of these was the Nurses' Health Study II, a prospective study of North American registered nurses aged 25–42 years at recruitment (Eliassen *et al*, 2009, 2012). The second was a population-based study of incident breast cancer among women of Asian ancestry living in California and Hawaii (Faupel-Badger *et al*, 2010) and the third was a randomised trial of the effect of consuming soy foods on hormonal outcomes, conducted in women of Caucasian, Native Hawaiian and Asian ancestry, and living in Hawaii (Maskarinec *et al*, 2012).

Comparing absolute levels of EMs in our study with those reported by these other studies is not straightforward. Levels of individual EMs and all EMs combined may differ between ethnicities (Maskarinec *et al*, 2012) and women from several different ethnicities have been included in the reports to date (Asian, Faupel-Badger *et al* (2010); African-American, Asian, Hispanic and Caucasian, Eliassen *et al* (2012); Caucasian, Native Hawaiian and Asian, Maskarinec *et al* (2012)). In addition, the 30 non-carriers that we analysed may not be representative of the general British population, as they were selected on genotype (with a MAF of 4%, we would expect 2.2 CYP3A7*1C carriers in a population-based sample of 30 women) and 6 (20%) were first-degree relatives of breast cancer cases, suggesting that they may be at higher risk than the general population. Our analysis is based on the average of two periovulatory samples, Faupel-Badger *et al* (2010) analysed both non-luteal phase and luteal phase samples, whereas the studies reported by Eliassen *et al* (2009, 2012) and Maskarinec *et al* (2012) focussed on luteal phase samples. There is, however, no evidence that the 2-OHE_1_:16α-OHE_1_ ratio differs according to the phase of the menstrual cycle (Faupel-Badger *et al*, 2010). Comparing this ratio across studies, the 2-OHE_1_:16α-OHE_1_ ratio in our non-carriers (3.9) was similar to the 2-OHE_1_:16α-OHE_1_ ratios reported by Faupel-Badger *et al* (2010) (2.2 and 2.1 in luteal phase and non-luteal phase samples, respectively) and by Eliassen *et al* (2012), (4.35) but somewhat lower than the ratios reported by Maskarinec *et al* (2012) (8.4 and 13.0 in Asian and non-Asians, respectively).

We have previously demonstrated that the CYP3A7*1C allele is associated with lower levels of urinary E_1_G (Johnson *et al*, 2016). Here we have demonstrated that CYP3A7*1C carriers have a more pronounced reduction in 2-hydroxylation pathway EMs, increased 16α-hydroxylation pathway EMs and markedly reduced two-hydroxylation pathway:16α-hydroxylation pathway ratios as measured by the 2-OHE_1_:16α-OHE_1_ ratio (0.39 in CYP3A7*1C carriers compared with population estimates of 2.1–13.0) or all 2- and 16α-pathway metabolites combined (0.10 in CYP3A7*1C carriers compared with population estimates of 0.90 or 0.96 (Eliassen *et al*, 2009, 2012)). Our data are consistent with expression of the foetal *CYP3A7* gene in adult carriers of the CYP3A7*1C allele resulting in (i) a modest reduction in levels of the parent oestrogen E_1_ and (ii) a specific bias towards 16α-hydroxylation (which can be catalysed by CYP3A7) over 2- or 4- hydroxylation (which are not catalysed by CYP3A7 (Lee *et al*, 2003)).

In their recent review of oestrogen metabolism and breast cancer, Ziegler *et al* (2015) concluded that the combined evidence from prospective studies using LC-MS to measure EMs in pre-diagnostic serum, plasma and urine was consistent with the hypothesis that enhanced 2-hydroxylation is associated with reduced risk of breast cancer. They further concluded that the inverse association with enhanced 2-hydroxylation (specifically 2-hydroxylation:16α-hydroxylation and 2-hydroxylation:parent oestrogens ratios) was independent of the strong positive associations between unconjugated E_2_ and E_1_ with postmenopausal breast cancer risk (Ziegler *et al*, 2015). Our analysis supports these conclusions if we assume that breast cancer risk in carriers of the CYP3A7*1C allele is influenced by two components with opposite effects. Based on the combined evidence of prospective studies of premenopausal hormone levels and breast cancer risk, lifetime lower levels of E_1_ and E_2_ of ∼45% and 27% in carriers of the CYP3A7*1C allele would be predicted to result in a substantial reduction in risk for these individuals. By contrast, an unfavourable (reduced) 2-hydroxylation:16α-hydroxylation ratio would be expected to increase risk. Our previous analyses demonstrate that the reduction in breast cancer risk for carriers of the rare allele of rs10235235 (which is correlated with the CYP3A7*1C allele *r*^2^=0.25, *D*'=1.0) is very modest (heterozygote OR=0.98, *P*=0.2; homozygote OR=0.80, *P*=0.004; Johnson *et al*, 2014) consistent with an unfavourable (reduced) 2-hydroxylation:16α-hydroxylation counteracting a potentially more substantial beneficial effect of lower levels of parent oestrogens.

Strengths of our study include our comprehensive analysis of urinary EMs using HPLC-MS/MS and, as genotypes are effectively randomised at birth (Ebrahim and Davey Smith, 2008), our focus on a genetic variant (the CYP3A7*1C allele), which minimises the potential for confounding by unmeasured environmental factors. There are several limitations to our study; owing to the frequency of the CYP3A7*1C allele, the numbers are small and, given our selection procedure, the non-carriers may not be representative of the White British population. Our choice of using two consecutive periovulatory samples makes it difficult to compare EM levels in our study with other published reports, which have mainly analysed a single luteal phase sample (Eliassen *et al*, 2009, 2012; Faupel-Badger *et al*, 2010; Maskarinec *et al*, 2012). In addition, we do not have prospective data on CYP3A7*1C carrier status, hormone levels and breast cancer risk in large numbers of women, and we cannot compare breast cancer risk in women with low levels of the parent oestrogen E_1_ according to their CYP3A7*1C status (and hence 2-hydroxylation:16α-hydroxylation ratio) directly.

In conclusion, we have demonstrated that the CYP3A7*1C allele has a profound effect on levels of the parent oestrogen E_1_ and the ratio of 2-hydroxylation:16α-hydroxylation EMs in premenopausal women. To characterise the association between parent oestrogens, EMs and subsequent risk of breast cancer fully, identification of additional genetic variants that influence parent oestrogens and particular pathway EMs, and further prospective studies that analyse a broad spectrum of EMs will be required.

## Figures and Tables

**Figure 1:**
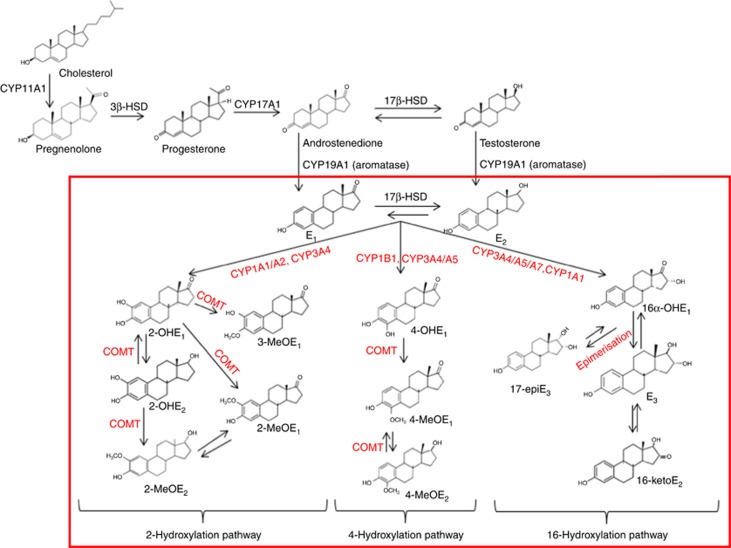
**Steroid hormone synthesis and endogenous oestrogen metabolism in humans.** The 14 oestrogen metabolites (EMs) formed by hydroxylation of parent oestrogens (E_1_ and E_2_), at the 2-, 4- and 16α-positions of the carbon ring that were measured are shown within the red box. Enzymes (cytochrome P450 (CYPs) and catechol O-methyltransferase (COMT)) involved in oestrogen metabolism are in red. E_1_G, measured in our previous analysis (Johnson *et al*, 2012), is an E_1_ conjugate present in urine. The E_1_ measured in this analysis (after hydrolysis of glucuronide and sulphate conjugates in the first step of the LC-MS/MS protocol) is highly correlated with E_1_G (Spearman's correlation, *ρ*=0.70, *P*<0.0001). Abbreviations: E_1_=oestrone; E_2_=estradiol; 2-OHE_1_=2-hydroxyestrone; 2-OHE_2_= 2-hydroxyestradiol; 2-MeOE_1_=2-methoxyestrone; 2-MeOE_2_=2-methoxyestradiol; 3-MeoE_1_=2-hydroxyestrone-3-methyl ether; 4-OHE_1_=4-hydroxyestrone; 4-MeOE_1_=4-methoxyestrone; 4-MeOE_2_=4-methoxyestradiol; 16α-OHE_1_=16α-hydroxyestrone; E_3_=estriol; 16-KetoE_2_=16-ketoestradiol; 17-epiE_3_=17-epiestriol and 17*β*-HSD=17*β*-hydroxysteroid.

**Table 1:** Characteristics of the 30 CYP3A7*1C carriers and 30 CYP3A7*1C non-carriers included in this analysis

	CYP3A7*1C **non-carriers**	CYP3A7*1C **carriers**	***P*****-value**
Age at urine collection	44.1 (16–53)	45.5 (27–50)	0.38
BMI at urine collection	24.0 (19.2–39.9)	25.5 (18.3–39.0)	0.64
Parity			
Nulliparous	10 (33.3)	6 (20.0)	
1–2 Children	17 (56.7)	15 (50.0)	
>2 Children	3 (10.0)	9 (30.0)	0.13
Age at first full-term pregnancy^a^	26.3 (20–31)	27.2 (17–32)	0.39

Abbreviation: BMI=body mass index.

a In parous women. For quantitative traits (age at urine collection, BMI at urine collection and age at first full-term pregnancy), means and ranges are presented. For parity, the number and percentage of women in each category is shown. For quantitative traits, *P*-values were from *t-*tests; for parity, the *P*-value was from a Fisher's exact test.

**Table 2:** Percentage difference (95% CI) in urinary EM levels comparing CYP3A7*1C carriers with non-carriers

**Grouped EM**	**Individual EM**	**% Difference in EM**^a^,^b^ **(95% CI)**	***P*****-value**
Total EMs		−15.1 (−32.7, 7.0)	0.16
Parent EMs		−41.0 (−57.2, −18.5)	0.002
	Oestrone	−45.3 (−60.6, −24.1)	0.0005
	Estradiol	−26.7 (−47.5, 2.2)	0.07
Catechol EMs		−71.0 (−80.1, −57.9)	1.2 × 10^−8^
2-Catechol EMs		−76.1 (−83.0, −66.6)	8.4 × 10^−12^
	2-Hydroxyestrone^c^	−78.3 (−84.5, −69.6)	1.1 × 10^−12^
	2- Hydroxyestradiol	−67.9 (−78.3, −52.6)	2.7 × 10^−7^
4-Catechol EMs			
	4-Hydroxyestrone	−51.8 (−72.9, −14.2)	0.01
Methylated catechol EM			
Methylated 2-catechol EM		−73.5 (−81.2, −62.7)	1.9 × 10^−10^
	2-Methoxyestrone	−81.2 (−87.1, −72.6)	1.9 × 10^−12^
	2-Methoxyestradiol^c^	−62.8 (−78.9, −34.7)	0.0009
	2-Hydroxyestrone-3-methyl ether^c^	−21.9 (−50.3, 22.6)	0.28
Methylated 4-catechol EMs			
	4-Methoxyestrone	NA	NA
	4-Methoxyestradiol	NA	NA
2-Hydroxylation pathway EMs		−74.9 (−81.6, −65.8)	1.6 × 10^−12^
4-Hydroxylation pathway EMs		−51.8 (−72.9, −14.2)	0.01
16-Hydroxylation pathway EMs		34.7 (1.6, 78.5)	0.04
	16α-Hydroxyestrone	91.6 (20.2, 205.1)	0.007
	17-Epiestriol	160.1 (46.0, 363.4)	0.002
	Estriol	25.5 (−5.1, 66.0)	0.11
	16-Ketoestradiol^c^	62.4 (8.7, 142.7)	0.02

Abbreviations: CI=confidence interval; EM=oestrogen metabolite; NA=not available.

a Unadjusted analysis.

b A negative difference may be interpreted as lower levels in CYP3A7*1C carriers compared with non-carriers.

c Missing values: 2-OHE_2_, 1 sample (non-carrier); 2-MeOE_2_, 11 samples (11 women, 2 non-carriers, 9 carriers); 3-MeOE_1_, 11 samples (9 women, 2 non-carriers, 7 carriers), 16-ketoE_2_, 5 samples (3 women, 2 non-carriers, 1 carrier).

## References

[bib1] Badawi AF, Cavalieri EL, Rogan EG (2001) Role of human cytochrome P450 1A1, 1A2, 1B1, and 3A4 in the 2-, 4-, and 16alpha-hydroxylation of 17beta-estradiol. Metabolism 50(9): 1001–1003. 11555828 10.1053/meta.2001.25592

[bib2] Burk O, Tegude H, Koch I, Hustert E, Wolbold R, Glaeser H, Klein K, Fromm MF, Nuessler AK, Neuhaus P, Zanger UM, Eichelbaum M, Wojnowski L (2002) Molecular mechanisms of polymorphic CYP3A7 expression in adult human liver and intestine. J Biol Chem 277(27): 24280–24288. 11940601 10.1074/jbc.M202345200

[bib3] Ebrahim S, Davey Smith G (2008) Mendelian randomization: can genetic epidemiology help redress the failures of observational epidemiology? Hum Genet 123(1): 15–33. 18038153 10.1007/s00439-007-0448-6

[bib4] Eliassen AH, Spiegelman D, Xu X, Keefer LK, Veenstra TD, Barbieri RL, Willett WC, Hankinson SE, Ziegler RG (2012) Urinary estrogens and estrogen metabolites and subsequent risk of breast cancer among premenopausal women. Cancer Res 72(3): 696–706. 22144471 10.1158/0008-5472.CAN-11-2507 PMC3271178

[bib5] Eliassen AH, Ziegler RG, Rosner B, Veenstra TD, Roman JM, Xu X, Hankinson SE (2009) Reproducibility of fifteen urinary estrogens and estrogen metabolites over a 2- to 3-year period in premenopausal women. Cancer Epidemiol Biomarkers Prev 18(11): 2860–2868. 19843676 10.1158/1055-9965.EPI-09-0591 PMC2783292

[bib6] Faupel-Badger JM, Fuhrman BJ, Xu X, Falk RT, Keefer LK, Veenstra TD, Hoover RN, Ziegler RG (2010) Comparison of liquid chromatography-tandem mass spectrometry, RIA, and ELISA methods for measurement of urinary estrogens. Cancer Epidemiol Biomarkers Prev 19(1): 292–300. 20056650 10.1158/1055-9965.EPI-09-0643 PMC2836837

[bib7] Gonzalez FJ (1988) The molecular biology of cytochrome P450s. Pharmacol Rev 40(4): 243–288. 3072575

[bib8] Johnson N, De Ieso P, Migliorini G, Orr N, Broderick P, Catovsky D, Matakidou A, Eisen T, Goldsmith C, Dudbridge F, Peto J, Dos-Santos-Silva I, Ashworth A, Ross G, Houlston RS, Fletcher O (2016) Cytochrome P450 allele CYP3A7*1C associates with adverse outcomes in chronic lymphocytic leukemia, breast, and lung cancer. Cancer Res 76(6): 1485–1493. 26964624 10.1158/0008-5472.CAN-15-1410 PMC4795533

[bib9] Johnson N, Dudbridge F, Orr N, Gibson L, Jones ME, Schoemaker MJ, Folkerd EJ, Haynes BP, Hopper JL, Southey MC, Dite GS, Apicella C, Schmidt MK, Broeks A, Van't Veer LJ, Atsma F, Muir K, Lophatananon A, Fasching PA, Beckmann MW, Ekici AB, Renner SP, Sawyer E, Tomlinson I, Kerin M, Miller N, Burwinkel B, Marme F, Schneeweiss A, Sohn C, Guenel P, Truong T, Cordina E, Menegaux F, Bojesen SE, Nordestgaard BG, Flyger H, Milne R, Zamora MP, Arias Perez JI, Benitez J, Bernstein L, Anton-Culver H, Ziogas A, Clarke Dur C, Brenner H, Muller H, Arndt V, Dieffenbach AK, Meindl A, Heil J, Bartram CR, Schmutzler RK, Brauch H, Justenhoven C, Ko YD, Network G, Nevanlinna H, Muranen TA, Aittomaki K, Blomqvist C, Matsuo K, Dork T, Bogdanova NV, Antonenkova NN, Lindblom A, Mannermaa A, Kataja V, Kosma VM, Hartikainen JM, Chenevix-Trench G, Beesley J, kConFab I, Australian Ovarian Cancer Study GWu AH, Van den Berg D, Tseng CC, Lambrechts D, Smeets D, Neven P, Wildiers H, Chang-Claude J, Rudolph A, Nickels S, Flesch-Janys D, Radice P, Peterlongo P, Bonanni B, Pensotti V, Couch FJ, Olson JE, Wang X, Fredericksen Z, Pankratz VS, Giles GG, Severi G, Baglietto L, Haiman C, Simard J, Goldberg MS, Labreche F, Dumont M, Soucy P, Teo S, Yip CH, Phuah SY, Cornes BK, Kristensen VN, Grenaker Alnaes G, Borresen-Dale AL, Zheng W, Winqvist R, Pylkas K, Jukkola-Vuorinen A, Grip M, Andrulis IL, Knight JA, Glendon G, Mulligan AM, Devillee P, Figueroa J, Chanock SJ, Lissowska J, Sherman ME, Hall P, Schoof N, Hooning M, Hollestelle A, Oldenburg RA, Tilanus-Linthorst M, Liu J, Cox A, Brock IW, Reed MW, Cross SS, Blot W, Signorello LB, Pharoah PD, Dunning AM, Shah M, Kang D, Noh DY, Park SK, Choi JY, Hartman M, Miao H, Lim WY, Tang A, Hamann U, Forsti A, Rudiger T, Ulmer HU, Jakubowska A, Lubinski J, Jaworska-Bieniek K, Durda K, Sangrajrang S, Gaborieau V, Brennan P, McKay J, Slager S, Toland AE, Vachon C, Yannoukakos D, Shen CY, Yu JC, Huang CS, Hou MF, Gonzalez-Neira A, Tessier DC, Vincent D, Bacot F, Luccarini C, Dennis J, Michailidou K, Bolla MK, Wang J, Easton DF, Garcia-Closas M, Dowsett M, Ashworth A, Swerdlow AJ, Peto J, dos Santos Silva I, Fletcher O (2014) Genetic variation at CYP3A is associated with age at menarche and breast cancer risk: a case-control study. Breast Cancer Res 16(3): R51. 24887515 10.1186/bcr3662 PMC4522594

[bib10] Johnson N, Fletcher O, Naceur-Lombardelli C, dos Santos Silva I, Ashworth A, Peto J (2005) Interaction between CHEK2*1100delC and other low-penetrance breast-cancer susceptibility genes: a familial study. Lancet 366(9496): 1554–1557. 16257342 10.1016/S0140-6736(05)67627-1

[bib11] Johnson N, Walker K, Gibson LJ, Orr N, Folkerd E, Haynes B, Palles C, Coupland B, Schoemaker M, Jones M, Broderick P, Sawyer E, Kerin M, Tomlinson IP, Zvelebil M, Chilcott-Burns S, Tomczyk K, Simpson G, Williamson J, Hillier SG, Ross G, Houlston RS, Swerdlow A, Ashworth A, Dowsett M, Peto J, Dos Santos Silva I, Fletcher O (2012) CYP3A variation, premenopausal estrone levels, and breast cancer risk. J Natl Cancer Inst 104(9): 657–669. 22472546 10.1093/jnci/djs156

[bib12] Key T, Appleby P, Barnes I, Reeves G, Endogenous H Breast Cancer Collaborative Group (2002) Endogenous sex hormones and breast cancer in postmenopausal women: reanalysis of nine prospective studies. J Natl Cancer Inst 94(8): 606–616. 11959894 10.1093/jnci/94.8.606

[bib13] Key TJ, Appleby PN, Reeves GK, Travis RC, Alberg AJ, Barricarte A, Berrino F, Krogh V, Sieri S, Brinton LA, Dorgan JF, Dossus L, Dowsett M, Eliassen AH, Fortner RT, Hankinson SE, Helzlsouer KJ, Hoff man-Bolton J, Comstock GW, Kaaks R, Kahle LL, Muti P, Overvad K, Peeters PH, Riboli E, Rinaldi S, Rollison DE, Stanczyk FZ, Trichopoulos D, Tworoger SS, Vineis P (2013) Sex hormones and risk of breast cancer in premenopausal women: a collaborative reanalysis of individual participant data from seven prospective studies. Lancet Oncol 14(10): 1009–1019. 23890780 10.1016/S1470-2045(13)70301-2 PMC4056766

[bib14] Kuehl P, Zhang J, Lin Y, Lamba J, Assem M, Schuetz J, Watkins PB, Daly A, Wrighton SA, Hall SD, Maurel P, Relling M, Brimer C, Yasuda K, Venkataramanan R, Strom S, Thummel K, Boguski MS, Schuetz E (2001) Sequence diversity in CYP3A promoters and characterization of the genetic basis of polymorphic CYP3A5 expression. Nat Genet 27(4): 383–391. 11279519 10.1038/86882

[bib15] Lee AJ, Conney AH, Zhu BT (2003) Human cytochrome P450 3A7 has a distinct high catalytic activity for the 16alpha-hydroxylation of estrone but not 17beta-estradiol. Cancer Res 63(19): 6532–6536. 14559847

[bib16] Maskarinec G, Heak S, Morimoto Y, Custer L, Franke AA (2012) The relation of urinary estrogen metabolites with mammographic densities in premenopausal women. Cancer Epidemiol 36(5): e310–e316. 22537763 10.1016/j.canep.2012.03.014 PMC3410978

[bib17] Samavat H, Kurzer MS (2015) Estrogen metabolism and breast cancer. Cancer Lett 356(2 Pt A): 231–243. 24784887 10.1016/j.canlet.2014.04.018 PMC4505810

[bib18] Tsuchiya Y, Nakajima M, Yokoi T (2005) Cytochrome P450-mediated metabolism of estrogens and its regulation in human. Cancer Lett 227(2): 115–124. 16112414 10.1016/j.canlet.2004.10.007

[bib19] Walker K, Fletcher O, Johnson N, Coupland B, McCormack VA, Folkerd E, Gibson L, Hillier SG, Holly JM, Moss S, Dowsett M, Peto J, dos Santos Silva I (2009) Premenopausal mammographic density in relation to cyclic variations in endogenous sex hormone levels, prolactin, and insulin-like growth factors. Cancer Res 69(16): 6490–6499. 19679547 10.1158/0008-5472.CAN-09-0280

[bib20] Williams JA, Ring BJ, Cantrell VE, Jones DR, Eckstein J, Ruterbories K, Hamman MA, Hall SD, Wrighton SA (2002) Comparative metabolic capabilities of CYP3A4, CYP3A5, and CYP3A7. Drug Metab Dispos 30(8): 883–891. 12124305 10.1124/dmd.30.8.883

[bib21] Xu X, Keefer LK, Ziegler RG, Veenstra TD (2007) A liquid chromatography-mass spectrometry method for the quantitative analysis of urinary endogenous estrogen metabolites. Nat Protoc 2(6): 1350–1355. 17545972 10.1038/nprot.2007.176

[bib22] Xu X, Veenstra TD, Fox SD, Roman JM, Issaq HJ, Falk R, Saavedra JE, Keefer LK, Ziegler RG (2005) Measuring fifteen endogenous estrogens simultaneously in human urine by high-performance liquid chromatography-mass spectrometry. Anal Chem 77(20): 6646–6654. 16223252 10.1021/ac050697c

[bib23] Ziegler RG, Fuhrman BJ, Moore SC, Matthews CE (2015) Epidemiologic studies of estrogen metabolism and breast cancer. Steroids 99(Pt A): 67–75. 25725255 10.1016/j.steroids.2015.02.015 PMC5722219

