# Supplementary material for: CYP3A7*1C allele is associated with reduced levels of 2-hydroxylation pathway oestrogen metabolites
Source: Br J Cancer. 2017 Jan 10;116(3):382–8. doi: 10.1038/bjc.2016.432 (PMC5294487; doi:10.1038/bjc.2016.432)
Supplement: Supplementary Table 1 [file bjc2016432x1.docx]

Supplementary Table 1: Multiple reaction monitoring (MRM) transitions monitored for the protonated [MH+] EM-Dansyl and d-EM-dansyl metabolite and their retention times*.

| **Transitions monitored** | | | | |
| --- | --- | --- | --- | --- |
| **Estrogen metabolites-Dansyl** | **Precursor ion [M+H^+^]**  **(m/z)** | **Product ion 1 [M+H^+^] (m/z)** | **Product ion 2 [M+H^+^] (m/z)** | **Retention time (minutes)** |
| Estrone (E_1_) | 504 | 171 | 156 | 44.10 |
| Estradiol (E_2_) | 506 | 171 | 156 | 47.00 |
| 2-Hydroxyestrone (2-OHE_1_) | 753 | 170 | 519 | 54.80 |
| 2-Hydroxyestradiol (2-OHE_2_) | 755 | 170 | 521 | 56.90 |
| 2-Methoxyestrone (2-MeOE_1_) | 534 | 171 | 156 | 40.60 |
| 2-Methoxyestradiol (2-MeOE_2_) | 536 | 171 | 156 | 43.80 |
| 2-Hydroxyestrone-3-methyl ether (3-MeOE_1_) | 534 | 171 | 156 | 37.10 |
| 4-Hydroxyestrone (4-OHE_1_) | 753 | 170 | 519 | 57.00 |
| 4-Methoxyestrone (4-MeOE_1_) | 534 | 171 | 156 | 43.40 |
| 4-Methoxyestradiol (4-MeOE_2_) | 536 | 171 | 156 | 45.70 |
| 16α-Hydroxyestrone (16a-OHE_1_) | 520 | 171 | 156 | 27.30 |
| 17-Epiestriol (17-epiE_3_) | 522 | 171 | 159 | 36.10 |
| Estriol (E_3_) | 522 | 171 | 156 | 27.40 |
| 16-Ketoestradiol (16-ketoE_2_) | 520 | 171 | 156 | 27.30 |
| D4-E_2_ | 510 | 171 | 156 | 47.30 |
| D3-E_3_ | 525 | 171 | 115 | 27.60 |
| D2-16-epiE_3_ | 524 | 171 | 156 | 34.70 |
| D5-2-MeOE_2_ | 541 | 171 | 156 | 43.90 |
| D5-2-OHE_2_ | 760 | 170 | 526 | 57.00 |

Based on the retention time, chemical and structural similarities the following d-EM were used as internal standards for their respective EM: d4E2 for E2 and E1; d3E3 for E3, 16-ketoE2 and 16α-OHE1; d2-16- epiE3 for 17-epiE3; d5-2-MeOE2 for 2-MeOE2, 4-MeOE2, 2-MeOE1, 4-MeOE1 and 3-MeOE1; d5-2- OHE2 for 2-OHE2, 2-OHE1 and 4-OHE1. Product ion 1 was used as the quantifier ion and product ion 2 as the qualifier ion for each of their respective compounds.
