# Supplementary material for: CYP3A7*1C allele is associated with reduced levels of 2-hydroxylation pathway oestrogen metabolites
Source: Br J Cancer. 2017 Jan 10;116(3):382–8. doi: 10.1038/bjc.2016.432 (PMC5294487; doi:10.1038/bjc.2016.432)
Supplement: Supplementary Table 2 [file bjc2016432x2.docx]

Supplementary Table 2: Intra- and inter-batch coefficients of variation (CVs) for 14 urinary EMs.

| **Individual and grouped EMs** | **Intra-batch CV (%)** | **Inter-batch CV (%)** |
| --- | --- | --- |
| **Parent EMs** |  |  |
| Estrone | 9 | 9 |
| Estradiol | 9 | 9 |
| **Catechol EMs** |  |  |
| **2-Catechol EMs** |  |  |
| 2-Hydroxyestrone | 7 | 10 |
| 2-Hydroxyestradiol | 9 | 6 |
| **4-Catechol EMs** |  |  |
| 4-Hydroxyestrone | 10 | 6 |
| **Methylated Catechol EMs** |  |  |
| **Methylated 2-catechol EMs** |  |  |
| 2-Methoxyestrone | 6 | 6 |
| 2-Methoxyestradiol | 7 | 7 |
| 2-Hydroxyestrone-3-methyl ether | 9 | 9 |
| **Methylated 4-Catechol EMs** |  |  |
| 4-Methoxyestrone | 10 | 13 |
| 4-Methoxyestradiol | 9 | 14 |
| **16-Hydroxylation pathway EMs** |  |  |
| 16α-Hydroxyestrone | 6 | 9 |
| 17-Epiestriol | 7 | 7 |
| Estriol | 10 | 10 |
| 16-Ketoestradiol | 6 | 9 |

Intra- and inter-batch CVs were calculated from quality control (QC) samples, with 3 QC samples per analytical batch in each of six independent consecutive batches. QC samples comprised 2ng/ml of EMs in estrogen-free (charcoal stripped) human urine.
