# Supplementary material for: CYP3A7*1C allele is associated with reduced levels of 2-hydroxylation pathway oestrogen metabolites
Source: Br J Cancer. 2017 Jan 10;116(3):382–8. doi: 10.1038/bjc.2016.432 (PMC5294487; doi:10.1038/bjc.2016.432)
Supplement: Supplementary Table 3 [file bjc2016432x3.docx]

Supplementary Table 3: Levels of individual and grouped EMs in 30 *CYP3A7*1C* carriers (AC) and 30 non-carriers (AA)

| Grouped EM | Individual EM | Geometric mean (95% CI)  (pmol/mg creatinine) | |
| --- | --- | --- | --- |
|  |  | AA | AC |
| Total EMs |  | 169.92 (143.25,201.56) | 144.25 (122.45,169.93) |
| Parent EMs |  | 36.67 (29.51,45.57) | 21.65 (16.89,27.75) |
|  | Estrone | 28.64 (23.09,35.54) | 15.67 (12.13,20.25) |
|  | Estradiol | 7.84 (6.2,9.9) | 5.74 (4.48,7.35) |
| Catechol EMs |  | 33.41 (25.64,43.53) | 9.69 (7.35,12.76) |
| 2-Catechol EMs |  | 26.39 (20.61,33.79) | 6.3 (4.96,8) |
|  | 2-Hydroxestrone | 21.75 (17.0,27.84) | 4.72 (3.71,6.01) |
|  | 2- Hydroxyestradiol | 4.36 (3.31,5.73) | 1.4 (1.04,1.87) |
| 4-Catechol EMs |  |  |  |
|  | 4-Hydroxyestrone | 5.57 (3.72,8.35) | 2.68 (1.75,4.12) |
| Methylated Catechol EM |  |  |  |
| Methylated 2-Catechol EM |  | 10.14 (8.08,12.73) | 2.16 (1.61,2.91) |
|  | 2-Methoxyestrone | 9.44 (7.5,11.87) | 1.78 (1.31,2.42) |
|  | 2-Methoxyestradiol | 0.67 (0.51,0.87) | 0.25 (0.15,0.42) |
|  | 2-Hydroxyestrone-3-methyl ether | 0.74 (0.55,1) | 0.58 (0.4,0.82) |
| Methylated 4-Catechol EMs |  |  |  |
|  | 4-Methoxyestrone | 0.10 (0.05,0.19) | n/a |
|  | 4-Methoxyestradiol | 0.42 (0.27,0.66) | n/a |
| 2-Hydroxylation pathway EMs | | 38.04 (30.19,47.93) | 9.54 (7.69,11.83) |
| 4-Hydroxylation pathway EMs | | 5.44 (3.69,8) | n/a |
| 16-Hydroxylation pathway EMs | | 75.48 (60.96,93.46) | 101.67 (83.84,123.28) |
|  | 16α-Hydroxyestrone | 6.64 (4.64,9.49) | 12.72 (9.29,17.40) |
|  | 17-Epiestriol | 0.31 (0.2,0.46) | 0.80 (0.52,1.22) |
|  | Estriol | 63.59 (51.52,78.49) | 79.81 (65.78,96.83) |
|  | 16-Ketoestradiol | 2.69 (1.96,3.69) | 4.36 (3.35,5.67) |
| Ratios |  |  |  |
| 2-OHE1/16α-OHE1 |  | 3.86 (2.53,5.89) | 0.39 (0.26,0.57) |
| 4-Catechol/2-Catechol |  | 0.22 (0.16,0.29) | 0.42 (0.30,0.58) |
| 2-Catechol/16-pathway |  | 0.35 (0.26,0.47) | 0.06 (0.04,0.09) |
| Catechol/16-pathway |  | 0.44 (0.32, 0.61) | 0.09 (0.07,0.14) |
| 2-pathway/16-pathway |  | 0.51 (0.38, 0.67) | 0.10 (0.07,0.13) |
| 2-Cathechols/methylated-2-catechols | | 2.63 (2.22, 3.11) | 3.20 (2.64,3.87) |
| Parent estrogens/estrogen metabolites | | 0.22 (0.18, 0.26) | 0.15 (0.12,0.18) |
|  |  |  |  |

* missing values: 2-OHE_2_, 1 sample (AA-genotype); 2-MeOE_2_, 11 samples (11 women, 2 AA, 9 AC); 3-MeOE_1_, 11 samples (9 women, 2 AA, 7 AC), 16-ketoE_2_, 5 samples (3 women, 2 AA, 1 AC).
