# Supplementary material for: CYP3A7*1C allele is associated with reduced levels of 2-hydroxylation pathway oestrogen metabolites
Source: Br J Cancer. 2017 Jan 10;116(3):382–8. doi: 10.1038/bjc.2016.432 (PMC5294487; doi:10.1038/bjc.2016.432)
Supplement: Supplementary Table 4 [file bjc2016432x4.docx]

Supplementary Table 4: Intra-class correlation coefficients for individual and grouped urinary EMs.

| **Individual and grouped EMs** | **ICCs (95% CI)** |
| --- | --- |
| **Total EMs** | 0.65 (0.41-0.87) |
| **Parent EMs** | 0.64 (0.38-0.85) |
| Estrone | 0.65 (0.39-0.86) |
| Estradiol | 0.65 (0.38-0.85) |
| **Catechol EMs** | 0.60 (0.38-0.81) |
| **2-Catechol EMs** | 0.70 (0.49-0.91) |
| 2-Hydroxyestrone | 0.71 (0.51-0.92) |
| 2-Hydroxyestradiol | 0.64 (0.40-0.79) |
| **4-Catechol EMs** |  |
| 4-Hydroxyestrone | 0.68 (0.45-0.81) |
| **Methylated 2-catechol EMs** | 0.85 (0.74-0.97) |
| 2-Methoxyestrone | 0.86 (0.75-0.97) |
| 2-Methoxyestradiol | 0.62 (0.32-0.83) |
| 2-Hydroxyestrone-3-methyl ether | 0.68 (0.40-0.86) |
| **Methylated 4-Catechol EMs** |  |
| 4-Methoxyestrone | n/a |
| 4-Methoxyestradiol | n/a |
| **2-Hydroxylation pathway EMs** | 0.73 (0.54-0.92) |
| **4-Hydroxylation pathway EMs** | n/a |
| **16-Hydroxylation pathway EMs** | 0.76 (0.59-0.94) |
| 16α-Hydroxyestrone | 0.70 (0.48-0.91) |
| 17-Epiestriol | 0.83 (0.70-0.96) |
| Estriol | 0.76 (0.58-0.93) |
| 16-Ketoestradiol | 0.80 (0.66-0.95) |

ICCs were calculated based on two consecutive periovulatory samples per woman.

For the majority of EMs estimates were based on measurements in 30 non-carriers,

exceptions were 2OHE2 (N=29), 2-MeOE_2_ (N=28), 3-MeOE_1_ (N=28), 16-ketoE_2_ (N=28).

n/a=not available
