# Supplementary material for: CYP3A7*1C allele is associated with reduced levels of 2-hydroxylation pathway oestrogen metabolites
Source: Br J Cancer. 2017 Jan 10;116(3):382–8. doi: 10.1038/bjc.2016.432 (PMC5294487; doi:10.1038/bjc.2016.432)
Supplement: Supplementary Table 5 [file bjc2016432x5.docx]

**Supplementary Table 5: Percentage difference (95% CI) in urinary EM levels comparing *CYP3A7*1C* carriers with non-carriers**

| Grouped EM | Individual EM | % Difference in EM^#^  (95% CI) | *P* |
| --- | --- | --- | --- |
| Total EMs |  | -9.4 (-31.1, 19.0) | 0.47 |
| Parent EMs |  | -38.2 (-57.2, -10.6) | 0.01 |
|  | Estrone | -43.1 (-61.1, -16.8) | 0.005 |
|  | Estradiol | -20.7 (-44.9, 14.0) | 0.20 |
| Catechol EMs |  | -71.9 (-81.5, -57.3) | 2.3 x 10^-7^ |
| 2-Catechol EMs |  | -78.1 (-84.8, -68.5) | 1.0 x 10^-10^ |
|  | 2-Hydroxyestrone* | -80.2 (-86.2, -71.5) | 1.9 x 10^-11^ |
|  | 2- Hydroxyestradiol | -69.3 (-80.3, -52.0) | 3.1 x 10^-6^ |
| 4-Catechol EMs |  |  |  |
|  | 4-Hydroxyestrone | -49.1 (-73.7, -1.5) | 0.05 |
| Methylated Catechol EM |  |  |  |
| Methylated 2-Catechol EM |  | -78.1 (-85.1, -67.9) | 4.1 x 10^-10^ |
|  | 2-Methoxyestrone | -80.4 (-86.5, -71.3) | 4.4 x 10^-11^ |
|  | 2-Methoxyestradiol* | -66.5 (-82.1, -37.1) | 0.001 |
|  | 2-Hydroxyestrone-3-methyl ether* | -26.1 (-56.1, 24.4) | 0.25 |
| Methylated 4-Catechol EMs |  |  |  |
|  | 4-Methoxyestrone | n/a | n/a |
|  | 4-Methoxyestradiol | n/a | n/a |
| 2-Hydroylation pathway EMs | | -76.3 (-82.9, -67.1) | 2.0 x 10^-11^ |
| 4-Hydroylation pathway EMs | | -47.4 (-72.5, 0.8) | 0.05 |
| 16-Hydroylation pathway EMs | | 52.1 (9.6, 111.0) | 0.01 |
|  | 16α-Hydroxyestrone | 116.7 (28.6, 265.4) | 0.005 |
|  | 17-Epiestriol | 209.9 (65.9, 478.8) | 0.0007 |
|  | Estriol | 41.5 (2.8, 94.8) | 0.03 |
|  | 16-Ketoestradiol* | 100.2 (30.2, 207.7) | 0.002 |

^#^ adjusted for measurement batch, age at first full term pregnancy, parity and body mass index

* missing values: 2-OHE_2_, 1 sample (non-carrier); 2-MeOE_2_, 11 samples (11 women, 2 non-carriers, 9 carriers); 3-MeOE_1_, 11 samples (9 women, 2 non-carriers, 7 carriers), 16-ketoE_2_, 5 samples (3 women, 2 non-carriers, 1 carrier).
